# Paediatric Behçet’s disease: a UK tertiary centre experience

**DOI:** 10.1007/s10067-016-3187-z

**Published:** 2016-02-01

**Authors:** Sira Nanthapisal, Nigel J Klein, Nicola Ambrose, Despina Eleftheriou, Paul A. Brogan

**Affiliations:** 1 Infection, Inflammation and Rheumatology Section, UCL Institute of Child Health and Great Ormond Street Hospital NHS Foundation Trust, 30 Guilford Street, London, WC1N 1EH UK; 2 Department of Rheumatology, University College London Hospital NHS Foundation Trust, London, UK

**Keywords:** Behçet’s disease, Children, Clinical features, Diagnostic criteria, Vasculitis

## Abstract

There are currently limited data regarding paediatric Behçet’s disease (BD), particularly in the UK. We describe the clinical spectrum, treatment and outcome of BD, and explore the relative sensitivities of the criteria for the diagnosis of BD in a UK paediatric cohort. Single retrospective case note review of children with a clinical diagnosis of BD presenting between 1987 and 2012. Demographics, clinical features, treatment and outcomes were recorded. The sensitivities of the International Study Group (ISG) and International Criteria for BD (ICBD) criteria were explored. BD disease activity was calculated using the Behçet’s Disease Activity Index (BDAI). Forty-six patients (22 male) were identified. Median age of onset was 4.87 (0.04–15.71) years; median time to diagnosis was 3.74 (0.25–13.48) years. Clinical features were recurrent oral ulceration (97.8 %), recurrent genital ulceration (73.9 %), gastrointestinal (58.7 %), musculoskeletal (47.83 %), cutaneous (23.9 %) involvement and uveitis (2 %). Recurrent genital ulceration was more common in female patients (*P* = 0.044). Thirty-seven patients (80.4 %) fulfilled the ICBD criteria; only 12 patients (26.1 %) fulfilled the ISG criteria. BDAI score at diagnosis was 7/20 (0–10/20) and significantly decreased to 5/20 (0–9/20) (*P* < 0.0001) at latest follow-up. The commonest systemic treatment was colchicine (76.1 %); anti-TNFα treatment was reserved for severe cases (15.5 %). Paediatric BD in the UK may present very early in life, sometimes with a family history, and with a low incidence of ocular involvement. Diagnostic delay is common. The majority of our patients required systemic therapy; anti-TNFα was reserved for severe cases and has largely superseded the use of thalidomide.

**Electronic supplementary material:**

The online version of this article (doi:10.1007/s10067-016-3187-z) contains supplementary material, which is available to authorized users.

## Introduction

Behçet’s disease (BD) [1] is a chronic multisystem vasculitis characterized by recurrent oral and genital ulceration, uveitis, cutaneous, articular, neurological and gastrointestinal involvement [2–8]. There are currently no universally recognised pathognomonic tests or biomarkers for BD disease activity, and thus diagnosis and disease monitoring require astute clinical acumen [9]. Although the usual onset of disease is between the second and fourth decade, there has been an increased awareness of BD during childhood [10–12]. Children account for 7.7–26.4 % of the reported prevalent cases of BD outside the UK [1, 3]. In one UK study, only one paediatric BD case was identified in a population of 1.1 million children [13]. Thus, due to the rarity of the condition and its non-specific presentation, the diagnosis of BD is challenging for paediatricians, and diagnostic delay of several years is common. This may lead to irreversible complications such as blindness, neurological injury and other end-organ damage.

There are several sets of criteria for the diagnosis and/or classification of BD [9]; none have been validated in children. The most widely used for adult onset disease are the International Behçet’s Study Group (ISG) criteria (Supplementary Table 1); these are considered to have suboptimal sensitivity for the classification or diagnosis of BD in adults, however [14]. Recently, International Criteria for Behçet’s Disease (ICBD) have been validated in adults (Supplementary Table 2) [7] with 94.8 % sensitivity and 90.5 % specificity. In comparison, the ISG criteria are 81 % sensitive and 96 % specific for the diagnosis of BD [7]. It is suggested therefore that the ICBD criteria should be used as the gold standard [7, 9]. There are currently no data in relation to the performance of the ICBD criteria in paediatric patients.

There is also a lack of evidence base for treatment of BD, particularly in children [15]. Notably, the vast majority of published paediatric series come from countries where BD is prevalent; consequently, the clinical phenotype and burden of disease is not well described for the UK-based paediatric population, and is likely to differ given established differences in genetic background and environment.

The primary aim of this study was to describe the clinical spectrum, therapy and outcome of children with BD presenting to a large tertiary paediatric referral centre based in the UK and compare with other international paediatric series. A secondary aim was to explore the relative sensitivities of the ISG and ICBD criteria for the diagnosis of BD in this paediatric cohort.

## Methods

### Study population

We identified from our clinical database, all patients (<18 years of age) with a clinical diagnosis of BD (based on clinicians’ expert opinion as the gold standard, see statistics section below) seen at Great Ormond Street Hospital in London between January 1987 and December 2012. Ethical approval was given by our Institution’s Research Ethics Committee for a retrospective case note review; thus, no written consent from patients was required. The demographic, clinical, and laboratory characteristics recorded at diagnosis were sex, age, ethnicity, organ involvement, erythrocyte sedimentation rate (ESR; reference range 0–10 mm/h) and serum C-reactive protein (CRP; reference range 0–20 mg/L). We tested the applicability of the ISG [2], and the ICBD criteria [7] in our cohort. The BD Activity Index (BDAI) score [16] (range 0–20, where 0 indicates no disease activity; full glossary of terms available as a supplementary document) was calculated retrospectively. The time from disease onset to diagnosis, number of relapses and relapse symptoms were recorded. Gender differences in presenting symptoms were explored. Disease and treatment-related morbidities were determined. Osteopenia was defined as a *z* score of body mass-adjusted bone density more than two standard deviations below the mean for age identified with a dual energy X-ray absorptiometry (DEXA) scan. Growth standard deviation scores (*z* scores) were calculated based on UK normative data using a free downloadable Microsoft Excel add-on [17]. Presenting clinical features and demographics of this UK cohort were compared to other published paediatric series.

### Statistical analyses

Continuous variables were summarised as median and range; categorical variables were presented as percentages. Parameters between groups were compared using Fisher’s exact test. *P* values <0.05 were considered significant. Statistical analysis was done with SPSS version 21. Estimates of sensitivity for the ISG and ICBD criteria were calculated using the formula: sensitivity = TP/(TP + FN); (true positive (TP); false-negative (FN), using physician diagnosis of BD as gold standard for the purposes of this retrospective study). 95 % confidence intervals for sensitivities were calculated. No estimates of specificity of either tool were obtained since this study did not include disease controls.

## Results

### Demographics and clinical characteristics

Forty-six patients were identified; 22 male (47.8 %). Eight patients (17.0 %) had a family history suggestive of BD in a first-degree relative. Forty-two patients (91.3 %) were Caucasian; two from United Arab Emirates; one Saudi Arabian; and one Palestinian. The median age of disease onset was 4.87 (0.04–15.71) years; median time to diagnosis was 3.74 (0.25-13.48) years, and median length of follow-up 2.54 (0.2–13.4) years (Supplementary Table 3).

#### Presenting features

Clinical features present within the first 6 months of disease onset were recurrent oral ulceration (OU; 82.6 %), recurrent genital ulceration (GU; 17.4 %), gastrointestinal symptoms (26.2 %), musculoskeletal symptoms (arthritis/arthralgia; 21.7 %), fever (28.3 %), skin lesions (10.9 %) and ocular involvement (2.2 %; Table 1).

**Table 1:** Summary of the clinical features present in first 6 months from diagnosis, and over the whole disease course in a cohort 46 children with Behçet’s disease in a single UK paediatric rheumatology tertiary centre

	Number (%) with feature in the first 6 months (*N* = 46)	Number (%) with feature in whole disease course (*N* = 46)
Recurrent oral ulceration	38 (82.6)	45 (97.8)
Genital ulceration	8 (17.4)	34 (73.9)
Skin lesions	5 (10.9)	11 (23.9)
Pustular lesions	1 (2.2)	3 (6.5)
Skin ulceration	1 (2.2)	2 (4.3)
Erythema nodosum	1 (2.2)	2 (4.3)
Necrotizing folliculitis	1 (2.2)	2 (4.3)
Acneiform lesions	1 (2.2)	2 (4.3)
Pathergy*	0	3/5 (60)
Ocular lesions	1 (2.2)	4 (8.7)
Anterior uveitis	1 (2.2)	2 (4.3)
Conjunctivitis	0	2 (4.3)
Gastrointestinal manifestations	12 (26.1)	26 (56.5)
Abdominal pain	4 (8.7)	12 (26.1)
Gastrointestinal haemorrhage	1 (2.2)	2 (4.3)
Gastrointestinal ulceration	2 (4.3)	7 (15.2)
Anal/perianal ulceration	2 (4.3)	5 (10.9)
Recurrent diarrhoea	3 (6.5)	6 (13.0)
Fever	13 (28.3)	14 (30.4)
Arthritis	4 (8.7)	10 (21.7)
Neurological manifestations	5 (10.9)	13 (28.3)
Seizure	0	1 (2.2)
Frequent headache	5 (10.9)	11 (23.9)
Vascular complication	2 (4.3)	3 (6.5)
Sagittal sinus thrombosis	1 (2.2)	2 (4.3)
Thrombophlebitis	0	1 (2.2)
Serositis		
Pericardial effusion	1 (2.2)	1 (2.2)

*Pathergy test was only performed in five patients, and was positive in 3/5

#### Cumulative clinical features

Recurrent OU was present in 45/46 (97.8 %) patients over the whole disease course. The median age of OU onset was 3.89 (0.04–15.71) years. Data on number of OU attacks per year was available for 40 patients and ranged from 4 to 12 (median 6) episodes/year. Genital ulceration was present in 34/46 patients (73.9 %) and commonly occurred after the onset of OU at the age of 10.5 (0.98–16) years with only 17.4 % children having genital ulceration in the first 6 months since disease onset. The scrotum was the most commonly affected area in males (13/13); vulval ulceration was the commonest affected genital site in females (19/21) followed by vaginal mucosal ulceration (2/21).

Fever affected 14/46 (30.4 %). Skin manifestations documented in 11 patients (23.9 %) included pustular lesions in 3/11, skin ulceration in 2/11, erythema nodosum in 2/11, necrotizing folliculitis in 2/11 and acneiform lesions in 2/11. A pathergy test was performed in five patients; 3/5 patients (all British Caucasian females) reported eruption of small pustules at the venipuncture sites 2–3 days later.

All patients had yearly ophthalmology reviews as a minimum. Ocular involvement was reported in only four patients (8.7 %): two male patients had anterior uveitis and two female patients had conjunctivitis, possibly unrelated to BD. For one patient, anterior uveitis was the first presenting symptom of BD. No patients had panuveitis or posterior uveitis.

Arthralgia was reported in 14 (30.4 %); arthritis in 10 patients (21.7 %) and myalgia in 4 cases (8.7 %).

Gastrointestinal involvement was present in 26 patients (56.5 %): transient severe abdominal pain (attributed to BD by the physician) was the most frequently reported finding (*n* = 12; 26.1 %), which regressed spontaneously or in response to corticosteroids and required no further investigation. Fourteen patients (30.4 %) had more serious gastroenterological pathology including gastrointestinal haemorrhage (melena or haematemesis) (*n* = 2) and recurrent anal/perianal ulcers in five patients (10.9 %). Gastrointestinal tract endoscopy was performed in 10 children and showed gastritis in 3 patients, colonic ulcers in 2 patients, oesophagitis in one patient and oesophageal ulceration in another. The remaining patients had a normal endoscopy.

Neurological involvement was documented in 13 patients (28.3 %); 1 patient developed seizures and 11 patients reported frequent (more than once a week) headaches. One patient gradually developed neurogenic bladder dysfunction and required suprapubic catheter insertion. Brain computed tomography scan and/or magnetic resonance imaging were done in six patients, and no abnormality was found in four patients. Sagittal sinus thrombosis was observed in the remaining two patients (4.3 %); this was the initial manifestation of their disease. Cerebral spinal fluid analysis was available for two patients and was normal.

Urinary sediment abnormalities included mild proteinuria (three cases; 6.5 %) and haematuria (two cases; 4.3 %). One patient had persistent microscopic hematuria; renal ultrasound was normal, and there was no evidence of biochemical disturbance to account for this.

Cardiac involvement was documented in one patient (2.2 %) who developed a mild pericardial effusion. Vascular complications were found in three patients (6.5 %): two with sagittal sinus thrombosis (as mentioned above) and one developed thrombophlebitis of the lower leg.

### Gender differences

Recurrent GU was significantly more common in females (*P = 0.044*); supplementary Table 3. There were no other significant gender differences observed for any other data. No data in relation to GU and onset of puberty were available.

### Laboratory evaluation

HLA-B51 typing was performed in three cases; negative in all. At diagnosis, ESR (measured in 42/46 patients) was 9 mm/h (range 1–122; 38 % had elevated ESR >10 mm/h); CRP (measured in 38) was 5 mg/L (range 1–60; only one patient had elevated CRP at presentation); white blood cell count was 6.92 × 1000 cells/mm^3^ (2.14–12.97); platelet count was 293.5 × 1000 cells/mm^3^ (179–570). Antinuclear antibodies were detected in 9/46 patients (19.6 %). One patient was transiently positive for proteinase 3 antineutrophil cytoplasmic antibodies (32 U/mL; normal <10 U/mL).

### Genetic testing for common autoinflammatory diseases

Genetic testing for autoinflammatory conditions became available in 2004 at our institution. 8/46 patients who had prominent fevers and/or systemic inflammation had testing for monogenic autoinflammation: mevalonate kinase deficiency (7/8), cryopyrin-associated periodic fever (4/8), familial Mediterranean fever (8/8) and NOD2 (1/8) mutations. All testing was negative for the above conditions.

### Performance of the ISG versus ICBD criteria for the diagnosis of BD

Retrospectively, only 6/46 patients fulfilled ICBD criteria at the first presentation, compared with 1/46 fulfilling the ISG criteria. At the time of formal clinical diagnosis 37/46 patients (80.4 %) fulfilled ICBD criteria compared with 8/46 patients (17.4 %) who fulfilled ISG criteria. During follow-up, four more patients presented more features thus fulfilling the ISG criteria. The sensitivities of both sets of diagnostic criteria at diagnosis and last follow-up are presented in Table 2.

**Table 2:** Retrospective application of the ICBD [7] and ISG [2] diagnostic criteria: sensitivity* of the criteria at diagnosis and last follow-up

		No. (%) of patients fulfilling the criteria (*N* = 46)	Sensitivity (%; 95 % confidence interval)
At diagnosis	ICBD	37 (80.4)	80 (67–93)
	ISG	8 (17.4)	17 (6.0–28)
At last follow-up	ICBD	37 (80.4)	80 (67–93)
	ISG	12 (26.1)	26 (13–39)

*Sensitivity = TP/(TP + FN); (true positive (TP); false-negative (FN), i.e. not fulfilling the criteria for a diagnosis of BD, using physician diagnosis of BD as gold standard for the purposes of this retrospective study). *ICBD* International Criteria for Behçet’s disease; *ISG* International Study Group

### Treatment

Our general therapeutic approach to immunosuppression was that of a ‘therapeutic ladder’, starting with topical corticosteroids, escalating sequentially to colchicine, systemic corticosteroids, followed by other drugs such as azathioprine, mycophenolate mofetil, thalidomide, or biologic therapy in cases of inadequate control [15].

Treatments received are summarised in Table 3. Topical therapies (received by all the patients) included singly or in combination corticosteroid mouthwashes or 0.1 % triamcinolone oral paste for oral lesions; and 1 % hydrocortisone cream for genital ulceration and other inflammatory cutaneous lesions. Forty-five patients (97.8 %) needed addition of systemic treatment a median of 4 months (0–28 months) from diagnosis. For first line systemic therapy, 24 patients (52.2 %) were treated with oral colchicine (dose ranging between 0.5 and 2 mg/day); 5 patients (10.9 %) received oral thalidomide (dose ranging between 1 and 2 mg/kg once weekly to daily [18]; all presenting before 2001; and 5 patients (10.9 %) with azathioprine (dose ranging between 1 and 3 mg/kg/day). Short-course oral prednisolone (dose 0.5–1 mg/kg/day for 7 days) was prescribed for relapsing oro-genital ulcers in six patients (13.0 %). Anti-tumour necrosis factor alpha (TNFα) was used in seven patients (15.2 %); adalimumab in five patients, infliximab in one patient and etanercept in one patient, at 19 (3.3–109) months after disease onset; 6/7 had severe gastrointestinal symptoms and one patient had relapsing genital ulcers refractory to other systemic medications. The median BDAI score prior to anti-TNFα was 8/20 (7–10); and significantly decreased to 5/20 (range 0–8) at latest follow-up (median 40, range 2–76 months; *P* = 0.0127. Regarding the two patients with sagittal vein thrombosis, one received warfarin for 6 months; the other only received low-dose aspirin since the thrombosis had probably occurred years previously as indicated by the presence of extensive venous collaterals on imaging. This patient ultimately required a ventriculoperitoneal shunt insertion due to intracranial hypertension that failed to respond to acetazolamide.

**Table 3:** Systemic treatment for 46 children with Behçet’s disease in a single UK paediatric rheumatology tertiary centre

	Total patients receiving treatment at any time (%)	First line systemic therapy no. (%) of patients
Colchicine (0.5–2 mg/day)	35 (76.1)	24 (52.2)
Thalidomide	13 (28.3)	5 (10.9)
Oral prednisolone	20 (45.5)	8 (17.3)
Short course^a^ (0.5–1 mg/kg/day)	6 (13.0)	6 (13.0)
Continuous^b^ (0.5–1 mg/kg/day)	14 (30.4)	2 (4.3)
Azathioprine	15 (32.6)	5 (10.9)
Sulfasalazine	3 (6.5)	1 (2.2)
Methotrexate	3 (6.5)	2 (4.3)
Adalimumab	5 (10.9)	–
Infliximab	1 (2.2)	–
Etanercept	1 (2.2)	–

^a^Short course oral prednisolone = a course of continuous oral prednisolone for <28 days

^b^Continuous oral prednisolone = continuous oral prednisolone >28 days

### Disease and treatment-related morbidities

Median BDAI at diagnosis was 7/20 (0–10) and significantly decreased to 5 (0–9; *P* < 0.0001) at latest follow-up of 2.54 years (0.2–13.4 years). Significant corticosteroid-related side effects included Cushing’s syndrome in 4/19 (21.1 %), and osteopenia in 2/19 patients (10.5 %) where this was assessed with DEXA scanning. Peripheral neuropathy was documented in 6/13 patients (46.2 %) receiving thalidomide therapy. Twenty-six patients had growth data available at the last follow-up; median height *Z* score was −0.49 (−2.41–2.51); median weight *Z* score was −0.1 (−2.33–2.74); and median body mass index (BMI) *Z* score was 0.28 (−2.23–2.37). No significant correlation between BDAI at diagnosis or last follow-up and growth *Z* score was found. One patient had a bone density *Z* score <−1.96 treated conservatively; no fractures were documented. One patient had ongoing neurogenic bladder symptoms at last review; and one aforementioned patient had a VP shunt. Of note, no patient had visual impairment; and no other permanent morbidities, or deaths occurred.

## Discussion

We describe the largest single-centre UK paediatric cohort of BD which suggests differences compared with studies involving Asian or Turkish children (Table 4). In addition, for the first time, we retrospectively examined the diagnostic sensitivity of the ICBD criteria in children. Patients in our study had early onset of disease at 4.87 years of age (median; range 0.04–15.71), and 17 % had a positive family history. The clinical presentation was varied and broad. Table 4 summarises the clinical phenotype of our UK-based paediatric cohort compared to other paediatric series [8, 19–22]. We observed less ocular involvement, and no posterior/panuveitis; less vascular disease; and less cutaneous disease. Gastrointestinal and neurological involvement was more frequent compared to non-UK cohorts. We noted a significant diagnostic delay up to 13.5 years. This is unfortunately common in children with BD [19], due to the insidious nature of the disease and an overall lack of awareness of BD. These differences could impact on choice of immunomodulatory therapy used since.

**Table 4:** Comparison of present UK study cohort to other published studies on childhood Behçet’s disease

Study (reference; country)	No. of patients	Male:female ratio	Median age of onset (year)	Percentage of patients in the series with each clinical features								
				OU (%)	GU (%)	Skin (%)	Ocular (%)	Pathergy (%)	Arthralgia/arthritis (%)	GI (%)	NS (%)	Vascular (%)
Present study	46	1:1.09	4.87	97.8	74	32.6	8.7	60^a^	52	58.7	32.6	6.5
Koné-Paut et al. (21; International)	110	1:0.96	8.2	83	61	57	33.9	44.7	N/A	26	29.3	13
*P* value		0.8608		0.0082	0.1434	0.0081	0.0012	0.6569		0.0002	0.7038	0.3984
Kitaichi et al. (20, Japan)	135	1:0.53	11.7	94.8	53.5	67.4	29	N/A	N/A	N/A	N/A	N/A
*P* value		0.0537		0.6816	0.0157	0.0001	0.0048					
Kim et al. (19; Korea)	40	1:1.5	10.6	100	82.5	72.5	27.5	N/A	27.5	5	2.5	N/A
*P* value		0.5183		1.000	0.4370	0.0003	0.0259		0.0001	0.0278	0.0002	
Karincaoglu et al. (8; Turkey)	83	1:1.18	12.3	100	81.9	N/A	34.9	37	39.8	4.8	7.2	16.8
*P* value		0.8554		0.3566	0.3664		0.0013	0.3703	0.1980	0.0001	0.0003	0.1110
Lang et al. (22; Canada)	37	1:0.947	8.7	100	75	84	30	N/A	54	40	32	16
*P* value		0.8267		1.0000	1.0000	0.0001	0.0204		1.0000	0.1244	1.0000	0.1783

*P* values were calculated by Fisher exact test. *P* values less than 0.05 (two-sided) were considered significant

*OU* oral ulceration, *GU* genital ulceration, *GI* gastrointestinal, *NS* nervous system, *N*/*A* data not available

^a^Pathergy test was only performed in five patients, and was positive in 3/5

Interestingly, only 8.7 % of the children had ocular involvement in our series and none had sight threatening disease with posterior or panuveitis; this contrasts with the high frequency of 33.9 % reported in an international study [21] and 42 % in Israeli children [23]. We speculate whether this could arise from genetic differences between our predominantly Caucasian population and non-UK cohorts. We also noted an increased frequency of genital ulceration in female patients (Supplementary Table 3).

We demonstrated the limitations of the ISG diagnostic criteria: only 17.4 % of our patients met these criteria at presentation to our centre. Similarly, in a previous multicentre paediatric study of BD, only 42 % fulfilled the ISG criteria as complete cases [21]; the authors proposed that age of puberty should be included as a parameter for definite BD as genital ulceration that usually starts after puberty; we were unable to assess this in our retrospective study since pubertal status was not assessed systematically. We demonstrated much higher sensitivity (80.4 %, 95 % CI 67–93 %; Table 2) of the ICBD diagnostic criteria compared to the ISG criteria at the time of clinical diagnosis. We did not study the specificity of the ICBD criteria, as this would require a disease control population comprising patients with important mimics of BD, such as periodic fever, aphthous stomatitis, pharyngitis and adenitis (PFAPA) amongst others (Supplementary Table 4). Whilst many experienced clinicians may not rely on such criteria for the diagnosis of BD in routine practice, such criteria are important for epidemiological and clinical studies. For instance, in the UK, a multi-centre collaborative study from the British Paediatric Surveillance Unit (BPSU) regarding the epidemiology and clinical phenotype of paediatric BD is now underway, and is using the ICBD criteria to define BD cases.

We explored the utility of the prototypic BDAI score for the first time in children in an attempt to systematically assess disease activity and response to therapy. We emphasise however, that retrospective scoring may be unreliable, and is therefore a limitation of our study. That said, this scoring system at least provides a systematic way of collecting such data retrospectively. BDAI scores significantly improved after treatment, and therefore could be an important tool for clinicians to monitor the BD disease course and should be now prospectively validated in paediatric patients.

Patients in our study were treated with a variety of therapeutic agents. The majority (93.5 %) of our patients required systemic therapy. Thalidomide-induced peripheral neuropathy occurred in 6/13 patients who received this; thus, less toxic therapeutic agents should be considered first. We used anti-TNFα treatment in severe/refractory cases (15.5 % of patients), an approach reported to be effective in adult studies [24]. More recently, IL-1 blockade has been highlighted as potentially useful [25], but we have not yet used this approach, nor interferon-α [26] for BD in children at our institution.

Overall, the prognosis for BD in our series was good. No child died; we did not observe any fractures; median final *Z* score for height, weight and body mass index at last follow-up was −0.49, −0.1 and 0.28, respectively. Despite these encouraging hard endpoints, the fact that 87 % of patients were still on treatment, emphasises that BD is a lifelong chronic disease.

Our study was limited by all the confounding factors associated with any single-centre retrospective series of patients with a rare disease. There is a possibility that referral bias resulted in us receiving the most severe cases. Pathergy and HLA B51 testing was not routinely performed. Importantly, we were unable to assess quality of life and any changes associated with treatment, an area of concern for patients with BD [27], and for understanding the heath economics of BD and its treatment in the young.

In summary, we present the largest UK-based single-centre series of children with BD, showing a distinctive clinical phenotype in this population. We observed less ocular involvement, less vascular disease, and less cutaneous disease. Gastrointestinal and neurological involvement was more frequent compared to non-UK cohorts. Colchicine appears to be an effective treatment; anti-TNFα treatment was used for refractory cases and has now largely superseded the use of thalidomide in our institution. Further prospective multicentre national studies using paediatric research networks such as the BPSU are currently underway, and will further characterise the epidemiology and clinical phenotype of BD in UK children, and could provide prospective data on whether any phenotypic differences influence choice of therapy. Lastly, whether these observed differences persist into adulthood remains to be determined, and will require long-term cohort studies. In the meantime, studies comparing children and adults with BD in the UK could help explore whether there are true differences between childhood and adult onset BD in the UK.

### Abbreviations

BD, Behçet’s disease; BDAI, Behçet’s disease activity index; BMI, body mass index; CRP, C-reactive protein, BPSU, British Paediatric Surveillance Unit; DEXA, dual energy X-ray absorptiometry; ESR, erythrocyte sedimentation rate; FN, false-negative; GU, genital ulceration; GI, gastrointestinal; HLA, human leukocyte antigen; ICBD, International Criteria for Behçet’s disease; IL, interleukin; ISG, International Study Group; NS, nervous system; OU, oral ulceration; PFAPA, periodic fever, aphthous stomatitis, pharyngitis and adenitis; TNF, tumor necrosis factor; TP, true positive; UK, United Kingdom; VP, ventriculo-peritoneal.

## Electronic supplementary material

Below is the link to the electronic supplementary material.

ESM 1 (DOCX 22 kb)
