# Supplementary material for: Paediatric Behçet’s disease: a UK tertiary centre experience
Source: Clin Rheumatol. 2016 Feb 1;35(10):2509–16. doi: 10.1007/s10067-016-3187-z (PMC5031738; doi:10.1007/s10067-016-3187-z)
Supplement: Supplementary file 1 — (DOCX 22 kb) [file 10067_2016_3187_MOESM1_ESM.docx]

**SUPPLEMENTARY DOCUMENTS**

**The Behçet’s disease activity index (BDAI)**

All scoring depends on symptoms present over the 4 weeks prior to assessment.

Only clinical features that the clinician feels are due to Behçet's Disease should be scored.

Ask the patient *"Over the past 4 weeks have you had any ………..?"* for the following items

1. Headache

2. Mouth ulceration

3. Genital ulceration

4. Erythema

5. Skin pustules

6. Joints - arthralgia

7. Joints - arthritis

8. Nausea, vomiting or abdominal pain

9. Diarrhoea plus altered or frank blood per rectum

10. New eye involvement: a red eye, a painful eye, blurred or reduced vision

11. New nervous system involvement (include intracranial vascular disease): Blackouts, difficulty with speech, difficulty with hearing, blurring of/double vision, weakness or loss of feeling of face, weakness or loss of feeling of arm, weakness or loss of feeling of leg, memory loss, loss of balance

12. New major vessel involvement (exclude intracranial vascular disease): Chest pain, breathlessness, coughed up blood, pain or swelling or discolouration of the face, pain or swelling or discolouration of the arm, pain or swelling or discolouration of the leg

**Scoring system**

Score = **1** for answering “YES” in each item. Sum up the score for the “patients index score” (0-12). Transform “patient’s index score” into “patient's Behçet’s Disease Activity Index Score” by using the table below.

| Patient’s index score | 0 | 1 | 2 | 3 | 4 | 5 | 6 | 7 | 8 | 9 | 10 | 11 | 12 |
| --- | --- | --- | --- | --- | --- | --- | --- | --- | --- | --- | --- | --- | --- |
| patient's Behçet’s Disease Activity Index Score | 0 | 3 | 5 | 7 | 8 | 9 | 10 | 11 | 12 | 13 | 15 | 17 | 20 |

**Reference**

Bhakta BB, Brennan P, James TE, Chamberlain MA, Noble BA, Silman AJ. Behcet's disease: evaluation of a new instrument to measure clinical activity. Rheumatology. 1999 Aug;38:728-33.

**Supplementary Table 1:** International Study Group criteria for diagnosis of Behçet’s disease

| Recurrent oral ulcerations | Minor aphthous, major aphthous or herpetiform ulceration observed by physician or patient, which recurred at least 3 times in one 12 month period |
| --- | --- |
| *Plus 2 of the following* |  |
| Recurrent genital ulceration | Aphthous ulceration or scarring observed by physician or patient |
| Eye lesions | Anterior uveitis, posterior uveitis, or cells in vitreous on slit lamp examination or retinal vasculitis observed by ophthalmologist |
| Skin lesions | Erythema nodosum observed by physician or patient, pseudofolliculitis or papulopustular lesions, or acneform nodules observed by physician in post-adolescent patients not on corticosteroid treatment |
| Positive pathergy test | Read by physician 24-48 hours |

**Reference**

Criteria for diagnosis of Behcet's disease. International Study Group for Behcet's Disease. Lancet 1990;335:1078-80.

**Supplementary Table 2**: International Criteria for Behçet’s Disease (ICBD) – score ≥ 4 required for diagnosis

| **Sign/symptom** | **Points** |
| --- | --- |
| Ocular lesions | 2 |
| Genital aphthosis | 2 |
| Oral aphthosis | 2 |
| Skin lesions | 1 |
| Neurological manifestation | 1 |
| Vascular manifestations | 1 |
| Positive pathergy test* | 1 |

**Ocular lesion**: anterior uveitis, posterior uveitis and/or retinal vasculitis; **skin lesions**: pseudofolliculitis (BD pustulosis), erythema nodosum and/or skin aphthosis; **neurological manifestation** includes peripheral and/or central symptoms with evidence of association with disease status; **vascular manifestations**: arterial thrombosis, large vein thrombosis, phlebitis and superficial phlebitis; ***Pathergy test** is optional.

**Reference**

International Team for the Revision of the International Criteria for Behcet's D. The International Criteria for Behcet's Disease (ICBD): a collaborative study of 27 countries on the sensitivity and specificity of the new criteria. J Eur Acad Dermatol Venereol 2014;28:338-47.

**Supplementary Table 3**: Comparison of presenting symptoms of Behçet’s disease between male and female group of patients in a UK based single paediatric rheumatology centre *

|  | **Total** | **Male** | **Female** | ***P value*** |
| --- | --- | --- | --- | --- |
| Number of cases (%) | 46 (100%) | 22 (47.8) | 24 (52.2) |  |
| Family history of Behçet’s (%) | 6 (13) | 4 (18.2) | 2 (8.3) | *0.4052* |
| Silk route Ancestry (%) | 8 (17.4) | 4 (18.2) | 4 (16.7) | *1* |
| Median age of onset (year; range) | 4.87 (0.04-15.71) | 4.9 (0.04-14.85) | 3.89 (0.23-15.71) | *0.8413* |
| Median age of diagnosis (year; range) | 11.12 (3.06-17.4) | 10.77 (3.28-17.4) | 11.35 (3.06-16.36) | *0.509* |
| Median time to diagnosis (year; range) | 3.74 (0.25-13.48) | 3.74 (0.48-11.18) | 4.15 (0.25-13.48) | *0.7385* |
|  |  |  |  |  |
| **Symptoms at diagnosis (%)** |  |  |  |  |
| Recurrent oral ulceration | 45 (97.8) | 21 (95.4) | 24 (100) | *0.4783* |
| Recurrent genital ulceration | 34 (73.9) | 13 (59.1) | 21 (87.5) | ***0.0440**** |
| Cutaneous lesions | 11 (23.9) | 8 (36.4) | 3 (12.5) | *0.0861* |
| Ocular involvement | 4 (8.7) | 2 (9.1) | 2 (8.3) | *1* |
| Positive pathergy | 3 (6.5) | 0 | 3 (12.5) | *0.2348* |
| Gastrointestinal involvement | 27 (58.7) | 13 (59.1) | 14 (58.3) | *1* |
| Arthritis/arthralgia | 22 (47.8) | 12 (54.6) | 10 (41.7) | *0.5552* |
| Fever | 14 (30.4) | 6 (27.3) | 8 (33.3) | *0.7539* |
| Neurological involvement | 11 (23.9) | 6 (27.3) | 5 (20.8) | *0.7343* |
| Vascular involvement | 3 (6.5) | 3 (13.6) | 0 | *0.1014* |
| Serositis | 1 (2.2) | 1 (4.6) | 0 | *0.4783* |
| Genitourinary involvement | 0 | 0 | 2 (8.3) | *0.4899* |

**P* values were calculated by Fisher Exact Test. *P* values less than 0.05 (2-sided) were considered significant

**Supplementary Table 4: Important mimics of Behçet's disease in the paediatric population**

| **Disorder** | **Diagnostic features, or test** |
| --- | --- |
| Periodic Fever Aphthous ulceration Pharyngitis and Adenitis syndrome (PFAPA) | Clinical diagnosis that tends to improve in the 2nd decade of life unlike BD, which persists; PFAPA not associated with genital ulceration. |
| Common variable immunodeficiency | Serum Immunoglobulin |
| Chronic granulomatous disease (both X-linked an autosomal recessive forms) | Nitroblue tetrazolium test (or equivalent) |
| Autoinflammatory diseases particularly mevalonate kinase deficiency | Genetic screening for MVK mutation |
| Inflammatory bowel disease including coeliac disease | Endoscopy; coeliac autoantibodies |
| Infections, particularly herpes simplex virus | Viral swab |
| Autoimmune disease, particularly systemic lupus erythematosus | Autoantibody profile, C3 and C4 |
| Pemphigus and related blistering skin diseases | Skin biopsy |
